# Marine Biotoxins and Seafood Poisoning

**DOI:** 10.3390/toxins11100558

**Published:** 2019-09-24

**Authors:** Pedro Reis Costa, António Marques, Jorge Diogène

**Affiliations:** 1 IPMA-Portuguese Institute for the Sea and Atmosphere, Av. Alfredo Magalhães Ramalho 6, 1495-165 Lisbon, Portugal; prcosta@ipma.pt (P.R.C.); amarques@ipma.pt (A.M.); 2 IRTA Institute of Agrifood Research and Technology, IRTA, Ctra. Villafranco, 5, 43540 Sant Carles de la Ràpita, Spain

Prevalence of marine biotoxins in seafood has been associated with increasing frequency, intensity, and duration of harmful algal blooms, and an increase of the geographical and temporal distribution of harmful algae. New and emerging biotoxins have been recurrently detected in regions where they were previously absent, raising challenges to the economic sustainability of seafood production in coastal areas and to consumer health safety. The economic burden to seafood producers caused by the closure of production areas and a possible feeling of insecurity from consumers urges researchers to improve available knowledge on toxin dynamics in marine organisms and the environment. Epidemiological studies are scarce and risk characterization is needed, particularly for emerging toxins. It is critical to enhance collaborative multi- and trans-disciplinary actions to introduce eco-innovative sustainable strategies to improve shellfish and fish safety. Strengthening industrial competitiveness is achievable by developing fast and reliable methods for marine biotoxin detection, and by implementing mitigation strategies. Innovative toxicological approaches for seafood safety evaluation are also required. The seven articles of this special issue address such research needs and are organized into three groups: (i) toxin dynamics and effects in marine organisms, (ii) development of detection methods for marine toxins, and (iii) toxin exposure and risks associated with the consumption of contaminated seafood.

Within the first group of articles, Alvarez et al. [1] reported an extreme event in southern Chile of a bloom of *Alexandrium catenella* that caused mass mortality of several marine invertebrate species, and resulted in accumulation of high levels (exceeding 100 times the regulatory limit for human consumption) of paralytic shellfish poisoning (PSP) toxins in clams. This study highlights the need for assessing toxin dynamics in shellfish under controlled conditions to better understand and foresee the impacts of harmful algal blooms. Andres et al. [2] fed green-lipped mussel (*Perna viridis*) with the toxic dinoflagellate *Alexandrium minutum* under controlled laboratory conditions to assess the dynamics of PSP toxin levels during accumulation and elimination phases. Barbosa et al. [3] investigated the interaction of ocean warming with fish (*Sparus aurata*) exposure to PSP toxins through contaminated mussels to assess physiological responses and changes in toxin accumulation. Tetrodotoxin (TTX), which has a mode of action comparable to PSP toxins, was characterized in the greater blue-ringed octopus *Hapalochlaena lunulata* from Okinawa, Japan [4].

Regarding the development and optimization of methods for toxin detection, Chen and colleagues [5] optimized clean-up procedures based on immunoaffinity column purification before mass spectrometry detection, providing an improved way to detect the amnesic shellfish poisoning toxin domoic acid (DA), in an array of matrices. Lefebvre et al. [6] describes a DA-specific antibody in the human serum and report DA-chronic exposure to certain groups of shellfish consumers. Finally, Hayashi et al. [7] investigated the combined effect of okadaic acid and mycotoxins that are considered emerging toxins in the marine environment, in human intestinal cell lines.
